# Serum Klotho (but not haplotypes) associate with the post-myocardial infarction status of older adults

**DOI:** 10.6061/clinics/2016(12)09

**Published:** 2016-12

**Authors:** Roberta S Paula, Vinícius C Souza, Wilcelly Machado-Silva, Bruno Ratier S Almeida, Andersen C Daros, Lucy Gomes, Aparecido P Ferreira, Ciro J Brito, Cláudio Córdova, Clayton F Moraes, Otávio T Nóbrega

**Affiliations:** I Universidade de Brasília (UnB), Brasília/, DF, Brazil; II Centro Universitário de Brasília (UNICEUB), Brasília/, DF, Brazil; III Universidade Católica de Brasília (UCB-DF), Brasília/, DF, Brazil; IV Faculdades Promove (ICESP), Brasília/, DF, Brazil; V Universidade Federal de Juiz de Fora (UFJF), Juiz de Fora/, MG, Brazil

**Keywords:** Biomarker, Cardiovascular Event, Vascular Disorder, Elderly, Infarction

## Abstract

**OBJECTIVES:**

The number of deaths from vascular diseases is incredibly high worldwide, and reliable markers for major events are still needed. The current cross-sectional study investigated the association of Klotho haplotypes and Klotho serum levels with classic risk factors and a clinical history of vascular events.

**METHODS:**

Clinical, anthropometric, biochemical and nutritional assessments were conducted with 168 older adults, complemented by genotyping (rs9536314 and rs9527025) and the detection of serum Klotho (ELISA).

**RESULTS:**

Klotho levels and haplotypes did not associate with most classic risk factors for vascular events, including markers such as C-reactive protein and homocysteine. A positive association was only found between Klotho levels and the previous occurrence of a myocardial infarction by both correlational (*p*=0.006) and variance analyses (*p*<0.001), and these associations were independent of the context.

**CONCLUSION:**

Our results suggest that serum Klotho is higher in individuals with a clinical history of myocardial infarction but not with a history of coronary artery disease or stroke. None of the Klotho haplotypes were associated with the variables investigated herein.

## INTRODUCTION

Globally, vascular diseases accounted for 17.5 million deaths in 2012. Coronary artery disease (CAD) is the single most frequent cause of death worldwide, accounting for 12.8% of all deaths (over 7 million), whereas cerebrovascular events accounted for 11.9% of the same total 1. As the major finding associated with vascular events, atherosclerosis is a chronic/progressive, focal process with multifactorial etiology (genetic and lifestyle) that co-occurs with systemic disorders that share hormonal and inflammatory determinants 2 that may have an early age of onset but only manifest later in life 3,4. Atherosclerosis occurs in more than 50% of the westernized adult population worldwide 5,6.

In this context, CAD is characterized by the narrowing of the walls of coronary arteries (stenosis), which reduces the blood flow to the heart muscle and is mostly caused by the accumulation of atheromatous plaques due to common risk factors such as poor diet, smoking, family history and genetic factors. As a possible outcome, a myocardial infarction (MI) damages the cardiac tissue, with a diagnosis set by the marked elevation of serum indicators (preferably troponin), accompanied by clinical aspects such as symptoms suggestive of ischemia, new Q waves on electrocardiography, or imaging evidence of viable myocardium or ventricular contractility losses 7.

Regarding cerebrovascular diseases, 90% result from lesions of the carotid arteries 8,9, which can lead to cerebral vascular accidents (strokes) due to the sudden closure or rupture of the arterial or venous vasculature of the brain 10. Regardless of whether the injury is hemorrhagic or ischemic (the latter is defined as an acute dysfunction in arterial territory 11), brain damage can result from an acute thrombotic event and can manifested as hemiplegia, aphasia or seizures 12. No serum marker is currently available to specifically predict the risk of the occurrence of vascular events in the brain.

The most important factors that are commonly assessed in clinical settings and are suggestive of vascular events in general include circulating levels of homocysteine and C-reactive protein (CRP), in addition to levels of lipoproteins and a few other markers. Both homocysteine and CRP tend to be present at high concentrations in individuals who are at increased risk of vascular events 13. Although CRP predicts CAD-related events independently of other risk factors 14-16, its levels can also be transiently increased over 2 to 3 weeks due to a serious infection, trauma or extra-cardiac ischemic event 17, which can compromise a safe clinical evaluation based on these markers. Additionally, circulating homocysteine is negatively correlated with the intake of vitamins as B6, B12 and folate 18-21 and is therefore nutritionally biased. Chronic renal failure may also interfere with breakdown of this biomarker, leading to hyperhomocysteinemia 22. Thus, the low specificity of the markers considered so far highlights the need for new, reliable biomarkers for vascular disorders 23, with studies in both clinical and experimental contexts taking place worldwide in search of candidates that are less susceptible to interference by associated comorbidities or lifestyle factors. However, many molecular aspects in the development of atherosclerosis, CAD, MI and stroke remain unknown, which reveals an open field for studies to identify new factors of great value for elaborating clinical procedures for primary prevention or assessing subsequent stages.

Thus, the Klotho protein is a potential marker for vascular events. Suppressing the Klotho gene in animal models causes extensive phenotypes similar to those of the aging phenotype, including atherosclerosis, ectopic calcification, infertility, skin atrophy and severe hypoglycemia 24, while its overexpression increases the total life span of guinea pigs by 20 to 30% 25. These phenotypes led to its unusual name, which is an allusion to the Greek goddess that spins the thread of life 24. The human Klotho gene, located on chromosome 13, can undergo alternative splicing in its third exon, generating a secretable form with signaling properties 26. The anchored Klotho protein is mostly present in the distal convoluted tubules of the kidneys and the choroid plexus of the brain but can be post-translationally processed and shed into the blood, with the free extracellular domain functioning as a hormone 27. One important physiological property attributed to circulating Klotho is the initiation of a pathway that inhibits insulin/IGF1 signaling 28. A moderate inhibition of insulin-like signaling is a conserved mechanism for life extension 29, with evidence in humans that indicates that the maintenance of average, physiological levels of the insulin-like growth factor-1 among very old individuals can contribute to a healthier endothelium and microcirculation integrity 30.

The Klotho gene bears six single nucleotide polymorphisms (SNPs) in exon 2 and flanking regions, all in perfect linkage disequilibrium, and two that result in substitutions with functional importance (F352V, rs9536314 and C370S, rs9527025), which are the notorious FC and VS haplotypes 31,32. The minor VS variant of the human Klotho gene is associated with an increased risk of vascular diseases 31,32.

The main aim of this study was to simultaneously investigate the association among serum levels of C-reactive protein, homocysteine and Klotho with classical risk factors and previous vascular events in a sample of elderly subjects, when considering covariates such as the usual intake of macronutrients and the common Klotho haplotypes.

## MATERIALS AND METHODS

### Subjects and study design

This report consists of cross-sectional analyses with data obtained from community-dwelling elderly women and men of the urban outskirts of the Brazilian Federal District, aged 60 or over, to whom health screenings (medical, nutritional and/or pharmacological) to assess their risk for vascular events was indicated, conforming a cohort study known as Prognosis and Therapeutics in Geriatrics (ProTeGer) in Brasília, Brazil. Participation was voluntary, and informed written consent was obtained from each subject. Data were collected from August 2011 to July 2014 during consultations in the Geriatric Service of the Catholic University Hospital.

For this analysis, the inclusion criteria were age ≥60 years, spontaneously seeking the service and to complying with the medical protocol. Exclusion criteria included carriers of autoimmune disease, chronic or recurrent infections, neoplastic diseases, chronic renal disease (creatinine clearance <25 mL/min/1.73m^2^) and/or having used anti-inflammatory drugs 30 days before clinical and biochemical tests.

This study was performed in accordance with the Declaration of Helsinki guidelines on good clinical practices, and the institutional ethical committee approved the study. No participants were receiving nutritional guidance when laboratory and clinical data were obtained.

### Clinical procedures

Each subject was required to undergo biochemical, anthropomorphic and clinical examinations prior to admission in this study.

The medical evaluation included the investigation of prior vascular events, including CAD, MI and stroke, based on clinical history reported by the patient and/or companion. All queries by the clinical practitioners that addressed prior events were conducted with the support of information already in the medical records or were based on complementary exams brought in by patient/companion to the consultations. A case was rendered as presenting CAD whenever a procedure of cardiac catheterization had been indicated by a professional elsewhere and had yielded findings compatible with coronary artery occlusion. MI was identified when the subject’s history included an admission to an emergency service with clinical signs and symptoms of acute infarction, as long as enzymatic [creatine kinase MB (CK-MB) and troponin] and electrocardiographic evaluations were recalled to support such endpoint. Compatible drug interventions, stenting or surgical revascularization, if recalled, also accounted for the diagnosis of MI. Finally, stroke was identified if the subject’s history or present state showed signs and symptoms of an event and brain computerized tomography image or cerebral angiogram was available to confirm diagnosis. The occurrence of any vascular event was computed only when independently declared in at least two medical visits. For our analysis, subjects were segregated as patients with or without previous vascular events.

The presence of comorbidities, such as high blood pressure, dyslipidemia and type II diabetes mellitus, was diagnosed based on the guidelines specific to each chronic condition, and the continued use of drugs to control these conditions was recorded. Practitioners of physical exercises were those subjects who reported performing 30 minutes or more of exercise at any level for at least four days a week 33, while the smoking habit was defined as consumption of >100 cigarettes over a lifetime 34.

Biochemical tests included the determination of serum total cholesterol (TC) and fractions, triglycerides (TGL), glycemia, glycated hemoglobin, insulin, thyroid-stimulating hormone (TSH), CRP, and homocysteine. At admission, venous blood samples were drawn into EDTA-containing tubes after a 12 h-overnight fasting period. Laboratory tests were performed according to routine clinical analyses with reagents from Boehringer Mannheim (Germany) and were processed in an AutoHumalyzer device (Human GMBH, Germany). The concentration of very low density lipoprotein cholesterol (VLDL-c) was determined by dividing TGL levels by 5, whereas the Friedewald equation was used to calculate low density lipoprotein cholesterol (LDL-c) levels by subtraction of both VLDL-c and high density lipoprotein cholesterol (HDL-c) from TC. Cases were evaluated as negative or positive to assemble categorical variables for metabolic disorders. Lipid categorization was performed according to NCEP ATP III 35, with each volunteer identified as a carrier (or not) of mixed hyperlipemia (TC ≥200 mg/dl, LDL-c ≥130 mg/dl and/or TGL ≥150 mg/dl). Current use of antihyperlipidemic drugs was considered in the definition of hyperlipidemia. Type 2 diabetes was defined according to reference values established by the American Diabetes Association (fasting glycemia ≥126 mg/dl) 36 or the current use of oral hypoglycemic drugs or insulin. Systolic and diastolic arterial blood pressure were measured as recommended by the VI Brazilian Guidelines for Arterial Hypertension 37. Patients with a systolic arterial blood pressure of ≥140 mmHg and/or a diastolic arterial blood pressure ≥90 mmHg, as well as those who were regularly taking antihypertensive drugs, were classified as hypertensive. The HOMA index was calculated based on the ratio of the product of fasting insulin (mU/L) and fasting glucose (mmol/L) by 22.5 38.

### Dietary evaluation

The present study evaluated the usual consumption of macronutrients by the elderly women and men. A non-consecutive three-day dietary record was completed that included one weekend day. To fill out the forms correctly, each patient received information from trained dieticians on the number and sizes of food portions. To ensure the completion of the dietary record, the nutrition staff provided either personal or telephone assistance.

Seven to ten days after being distributed, the forms were returned by patients during their next office visit, which was scheduled to check the accuracy of the records and to complete the information. The nutrient composition was analyzed with the Diet Pro software, version 5i (A.S. Sistemas, Brazil), adjusted for all available databases and complemented with a chemical composition table for Brazilian foods 39. Subjects whose records indicated the use of macronutrient-containing supplementary material were excluded from analyses.

After entry of dietary data, absolute intakes (mg) of carbohydrates, protein and lipids were individually calculated. Absolute intakes were converted into relative calories from these macronutrients in relation to total dietary intake, also in calories, yielding variables referred to as dietary intakes of carbohydrates (DIC), of lipids (DIL) and proteins (DIP). Total energy intake (TEI) and the macronutrient assessments were expressed as the mean daily intake based on the 3-day dietary records.

To increase the reliability of data, the Brazilian Portuguese version of the Mini-Mental State Examination (MMSE) 40 was used. Patients were excluded from the analysis based on the following scores according to educational levels: <13 for illiterate individuals <17 for individuals with 1-7 years of schooling, and <25 for individuals with eight or more years of formal education 41.

Waist circumference (WC) was measured at the midpoint between the last rib and the iliac crest during the individual’s expiration 42.

### Klotho Genotyping

A total of 10 ml of blood was collected by venipuncture into tubes containing heparin; plasma was obtained by refrigerated centrifugation (4°C) of 5 ml at 1,000 g for 15 min.

Plasma was aliquoted and immediately frozen at -20°C until testing. DNA extraction used 5 ml of blood by the salting out method 44. The determination of FC and VS haplotypes of the Klotho gene was performed as described by Arking et al. 31, with modifications. Briefly, both polymorphic sites F352V (T/G; rs9536314) and C370S (G/C; rs9527025) were amplified in the same DNA segment by polymerase chain reaction (PCR), with forward 5′ aggctcatgccaaagtctgg 3′ and reverse 5′ gtttccatgatgaactttttgagg 3′ primers. Amplification conditions consisted of hot start at 80°C for 1 min, followed by an initial denaturation at 94°C for 2 min; 36 cycles consisted of incubation at 94°C for 40 s, annealing at 60°C for 45 s and extension at 72° C for 50 s, and the process was completed at 72°C for 5 min. Amplification of the 505 pb products was confirmed by eletrophoresis in 1.6% agarose gels. When primer exhaustion was visually verified, the polymorphic points were identified by direct, automatized sequencing of amplification products in a 3130 DNA Analyzer system (Applied Biosystems, Foster City, CA, USA), using the manufacturer’s reagents and procedures. Sequencing reactions were performed using both forward and reverse primers for the PCR step. Sequencing was recorded as successful if a high quality sequence was obtained in at least one direction.

### Serum detection of Klotho

The Klotho protein concentration in serum was determined by ELISA (Enzyme-linked Immunosorbent Assay) using a specific kit produced by the BlueGene^®^ Biotech company (Shangai, China) with an analytic sensitivity of 0.1 ng/mL, according to the manufacturer's instructions. All samples were analyzed in duplicate, while standard curve points were produced in triplicate. Readings were generated using a BioTek Absorbance Reader, ELx800 model (Winooski, VT, USA). The intra-assay variation coefficients ranged from 3.1 to 9.5%, with all inter-assay variation coefficients below 5.0%.

### Statistical analysis

Student’s t-test was used to test the influence of Klotho haplotypes on continuous anthropometric, clinical and biochemical variables. Then, to evaluate the occurrence and strength of the association of circulating levels of Klotho and of other classic biomarkers (C-reactive protein and homocysteine) with prior vascular events, correlation coefficients were obtained between the investigated serum biomarkers and the continuous and categorical anthropometric, clinical and biochemical variables of potential confounding effects in the main model. The close-to-normal distribution of all continuous variables was assessed using the Kolmogorov-Smirnov test. The association between continuous variables was evaluated using the Pearson’s correlation test, whereas the involvement of a least one categorical variable in the model was assessed using Spearman’s counterpart, with men and women represented by 1 or 2 and the absence or presence of a feature represented by 0 or 1, respectively. Whenever an interaction was noticed, the association of the biomarkers with the vascular events was tested by means of partial correlation analyses with an adjustment for the confounding variable(s) or condition(s). In addition, raw concentrations of each biomarker were tested across individuals who had or had not experienced an acute MI (AMI) prior to the study onset using the Student’s t-test, with a sequential ANOVA (logistic regression model) used to confirm a significant association. When results are significantly different, the effect sizes (*d*) and respective confidence intervals (95% CIs) are presented. Stepwise linear multivariate regression analysis was performed to assess the extent to which serum concentrations of the biomarkers could explain the variability in occurrence of the vascular events.

All analyses were performed with the Statistical Package for the Social Sciences (SPSS) for Windows (version 17.0). For this study, the standard two-tailed threshold for significance (*p*≤0.05) was adjusted following the Bonferroni’s correction for multiple comparisons when a trait is tested across *k* independent variables (eg., for *k*=10 tests, α≤0.005).

## RESULTS

After clinical-laboratory and dietary assessments and the verification of the inclusion and exclusion criteria, the eligible sample for analyses was composed of 168 elderly patients with a mean age of 73.1 years. In terms of the F352V polymorphism, 138 (82.1%) of the subjects were FF homozygotes, whereas 27 (16.1%) had the FV genotype and 3 (1.8%) had the VV genotype. The exact same proportions were found for the CC, CS and SS genotypes, with firm correspondence between F and C carriage and between V and S presence in each individual genotype, which is consistent with a perfect linkage disequilibrium across these two SNPs in our sample. This finding is highly consistent with the assumption of Arking and colleagues, who stated that the identification of the F352V variation *per se* can be used as a surrogate for the determination of the whole haplotype for epidemiological purposes 43.

Table 1 presents the results of an inferential analysis of the results, which investigated the associations between the Klotho haplotype and the leading clinical-laboratory variables determined in the sample. In clinical terms, the sample characterization revealed that a relatively low proportion of subjects had previous vascular events, but there was a significant prevalence of metabolic disorders that was compatible with a profile that was eligible for primary prevention. The analysis showed no association of haplotypes with any of the basic variables. In addition, there were no differences in the prevalence of prior CAD, stroke and AMI cases among the haplotype groups.

The possibility of the association of clinical-laboratory and dietary variables with circulating Klotho levels was verified using correlation tests (Table 2), where traditional serum markers for vascular events (CRP and homocysteine) were considered. In this context, serum CRP and homocysteine levels were significantly influenced by gender, the average level of CRP was 87% higher among women [*p*=0.012; *d*=0.23 (0.1; 0.4)] and homocysteine levels were 34% higher in men [*p*=0.020; *d*=-0.25 (-0.4; -0.1)]. In addition, sedentary individuals exhibited a 75% higher mean serum CRP level [*p*=0.044; *d*=0.23 (0.1; 0.4)] compared with physically active individuals. Apart from these associations, our correlation analyses revealed no other significant association between the three serum biomarkers and any of the clinical, biochemistry and dietary features that were investigated. Therefore, Klotho levels do not appear to be influenced by demographic aspects, lifestyle factors or the comorbidities presented by patients in a context that was compatible with primary care settings. Accordingly, macronutrient intake was not significantly correlated with any of the serum markers that were considered.

**Figure 1:**
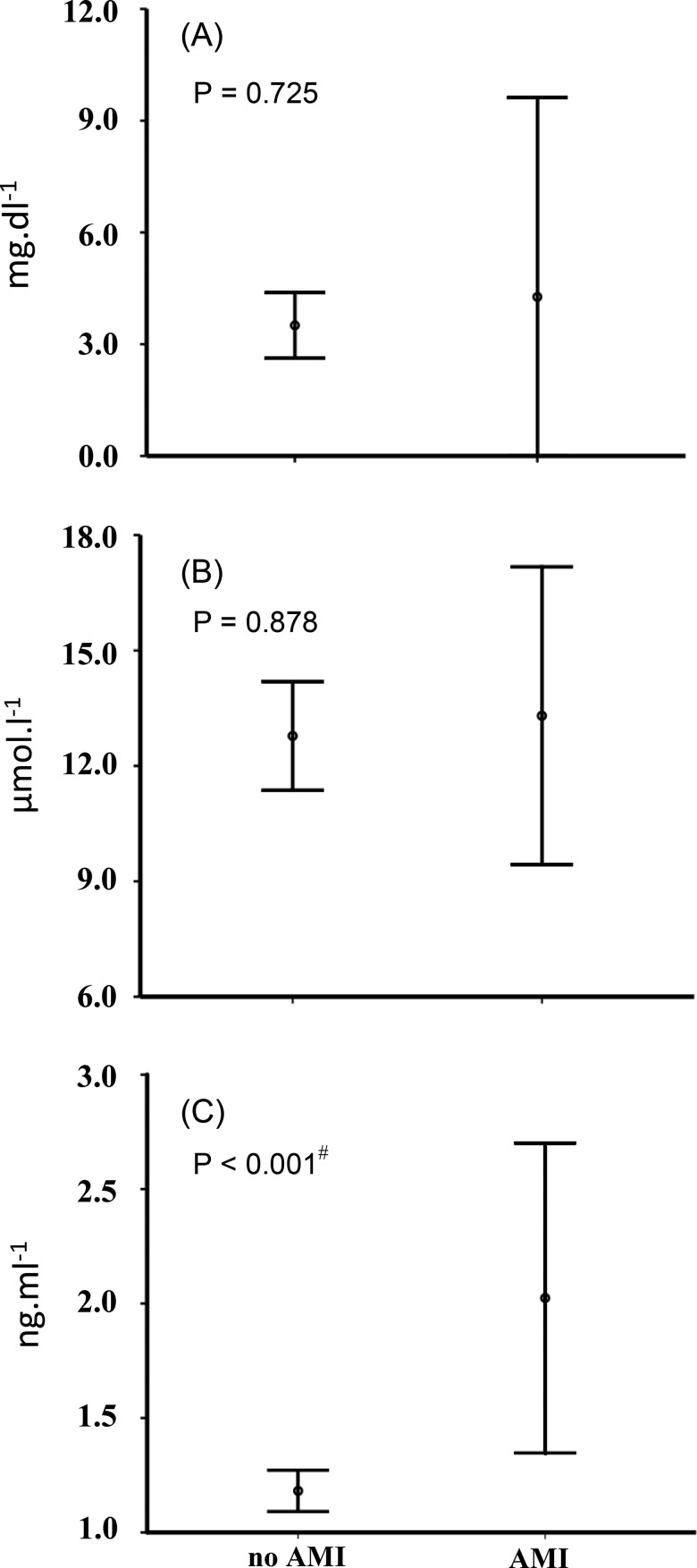
Comparisons of raw circulating levels of C-reactive protein (A), Homocysteine (B) and Klotho (C) in individuals who had or had not experienced a prior acute myocardial infarction (AMI). Significance was verified by Student’s *t-*test for independent samples and was confirmed by sequential ANOVA. Vertical bars represent intervals of one standard deviation. Superscript # represents an effect size of 1.6 and a 95% confidence interval of (0.9; 2.6).

Correlation analyses were then performed to investigate the association of CRP, homocysteine and Klotho biomarkers with the occurrence of previous vascular events (CAD, MI and stroke) at baseline (Table 3). These analyses were controlled for the presence of confounding factors (gender and physical inactivity) that had been previously associated with some biomarkers, and the main result consisted of a positive association between circulating Klotho levels and a prior AMI. When the serum levels of CRP, homocysteine and Klotho were compared among subjects, Klotho was the only marker to display different levels [72% higher among individuals affected by MI; *p*<0.001; *d*=1.6 (0.9 – 2.6)] according to a previous history of AMI (Figure 1). This association was confirmed by means of a sequential ANOVA with ten selected confounding factors of a discrete nature (age, WC, HOMA index, total cholesterol, triglycerides, SBP, DBP, TSH, CRP and homocysteine) that were tested alongside Klotho levels.

The analysis of multiple classic clinical, anthropometric and biochemical risk factors for vascular events along with circulating mediators via stepwise multivariate regression showed that the Klotho level was the single most predictive variable in the model (R^2^=0.086), and accounted for 29.3% of the variance in occurrence of AMI in the sample. At present, this result suggests an association and should be validated in another independent sample.

## DISCUSSION

Our study suggests that serum Klotho could be an independent marker for post-event MI among elderly individuals, with possible implications for secondary prevention, given the enhanced levels of the mediator among the affected outpatients. To our knowledge, this is the first report to identify this association. However, serum Klotho levels have been associated with different vascular risk factors in humans, such as atherosclerosis, oxidative stress and endothelial dysfunction 24,25,44,45. Most findings in the literature suggest a role for serum Klotho in the susceptibility to vascular, metabolic-born disorders. Intriguingly, our analyses on qualitatively and quantitatively portrayed metabolic traits (pressoric, lipemic, glycemic) of older adults across Klotho haplotypes or levels showed no significant associations. In addition, no associations were found in classic anthropometric measures, lifestyle features or health care practices. In our setting, this evidence suggests that Klotho does not relate to (and may not be an actual player in) the development of vascular disorders and events. Instead, we hypothesize that Klotho may be a previously unnoticed pathophysiological element that is linked to the cardiac post-MI state.

Ventricular dilation occurs during the acute phase (ventriculomegaly) of MI, and late cavitary dilation can occur due to eccentric hypertrophy 46,47. These dilations are adaptation processes in which the heart tends to assume a spherical shape due to the redistribution of forces for the maintenance of ventricular function as opposed to increased parietal stress (greater parietal tension in diastole than in systole) 48-50. Hypertrophied hearts in animal models show an increased expression of TRPC6 channels (transient receptor potential canonical 6), whose expression is regulated by different tensions and intensities. Soluble Klotho inhibits TRPC6 cardiac channels and thus protects the myocardium against excessive/pathological remodeling 51. Thus, high Klotho levels may appear post-infarction as an adaptation to this event in humans, supporting the finding of our study.

In favor of a rationale that supports the compensatory properties of Klotho after MI, adenovirus-mediated delivery of the gene into animal model ameliorates vascular endothelial dysfunction and prevents myocardial medial hypertrophy 44,52. However, no prior studies have associated serum Klotho levels in infarcted humans.

In addition to the functions already mentioned, Klotho can also act on the maturation of adipocytes and glucose metabolism. Klotho knockout rats are resistant to obesity even when exposed to a high fat diet 53,54, revealing a clear synergy between the soluble marker and diet. To control for these potential covariates, the total caloric intake and the proportional intake of macronutrients acquired from the usual diet were investigated in our study. However, our results indicate that there is no significant interaction between dietary profile and serum Klotho levels. Thus, the possibility that intake pattern was responsible for diet-related variations in the mediators was ruled out.

Although patients with major infarction-related myocardial remodeling show a progressive worsening of cardiac function, there is still no reliable marker for such a process, and slowing or reversing remodeling remains a challenge in the clinical care of post-MI subjects. However, few are the studies on new, reliable markers, and controversy (if any) may arise from the presence of confounding factors that were not adequately investigated or controlled. In this regard, our work has revealed that circulating Klotho levels did not vary under the influence of classic risk factors for vascular events (eg., glycemic and lipemic profiles), healthcare measures or demographic aspects like other biomarkers currently used in clinical practice (CRP and homocysteine). We were unable to rule out from our analysis subjects who consumed blood pressure-, glucose- or lipid-lowering drugs (>85% of the sample). However, our analyses demonstrated that these therapies did not alter any of the biomarkers that were investigated. There are limitations in our study that are worth mentioning. This was a cross-sectional study, and the fact that the clinical data relied mostly on the history of health events may lead to biases related to memory and the ability to recall. Temporal influence on the levels of serum markers cannot be discarded because the onset of the vascular events was not analyzed. In addition, the overall number of subjects was limited to detect small differences in the associations investigated. For example, our results did not show significant MI-related differences of CRP levels, a post hoc power analysis indicated that only a sample of 1116 individuals would suffice to provide power [*d*=0.20 (-0.19; 0:59)] to detect small differences in this variable. In terms of Klotho levels, it is outstanding that a reasonable effect size was reached in the correlational analysis [*d*=0.22 (0.1 – 0.4)] of our sample size of 168 subjects. Therefore, the results of our study could be interpreted as having fairly adequate power to detect meaningful differences.

Our results show that the serum Klotho concentration is higher in individuals with a clinical history of MI, but not in individuals with a history of CAD or stroke. Classic risk factors for vascular diseases were not associated either serum Klotho levels or the main genetic variations of Klotho. The augmented levels of Klotho post-infarction may reflect a compensatory mechanism to prevent pathological myocardial hypertrophy. However, further studies with an appropriate, prospective design are needed to evaluate the pathophysiological mechanisms of serum Klotho fluctuation in post-event settings.

## AUTHOR CONTRIBUTIONS

R.S. Paula and W. Machado-Silva performed the clinical anthropometric and pharmacological assessments, respectively.

L. Gomes and C.F. Moraes performed the medical component of the study.

V.C. Souza, A.C. Daros and B.R.S. Almeida participated in the laboratory assessments of Klotho genotypes and levels.

A.P. Ferreira, C.J. Brito, C. Córdova and O.T. Nóbrega designed and coordinated the study.

R.S. Paula and O.T. Nóbrega analyzed and interpreted the results and participated in the preparation of the original manuscript.

**Funding:** The research was supported with grants #471016/2011-0 (CNPq) and #193.000.032-2012 (FAPDF), with a stipend to W. Machado-Silva (CAPES) and a fellowship for productivity in research to O.T. Nóbrega (CNPq).

## Figures and Tables

**Table 1:** Anthropometric, clinical and metabolic variables of the sample.

	Groups			
Variables	All (n = 168)	FC/FC (n = 138)	___/VS (n = 30)	*p*
Male, %	39.9	37.7	50.0	0.212
Age, years	73.1±9.0	73.0±8.7	73.7±10.1	0.677
WC, cm	97.4±11.5	98.0±11.8	95.2±9.9	0.204
Glucose level, mg.dl^-1^	103.2±27.7	104.4±29.9	98.2±13.2	0.082
HbA1c, %	5.9±1.0	5.9±1.1	5.7±0.6	0.280
Insulin, mUI/mL	9.3±10.0	9.7±10.8	7.3±4.9	0.230
HOMA index	2.6±3.8	2.8±4.1	1.8±1.2	0.163
DM2^§^, %	22.6	26.1	6.7	0.021
Total cholesterol, mg.dl^-1^	193.4±39.9	192.3±36.2	198.8±50.1	0.404
LDL-c, mg.dl^-1^	115.3±33.7	114.4±33.1	119.6±37.0	0.448
Triglycerides, mg.dl^-1^	141.2±64.7	143.3±62.1	131.6±75.8	0.370
Hyperlipemia^§^, %	51.8	50.7	56.7	0.438
HDL-c, mg.dl^-1^	48.1±10.9	47.5±10.6	50.8±12.2	0.135
SBP, mm Hg	134.8±19.5	134.8±19.5	134.5±19.8	0.927
DBP, mm Hg	80.6±11.1	80.6±11.1	80.8±11.8	0.918
SAH^§^, %	77.4	78.3	73.3	0.559
CRP, mg.dl^-1^	3.5±5.6	3.7±5.8	2.6±4.3	0.339
TSH, mIU.l^-1^	2.5±2.2	2.6±2.3	2.2±1.6	0.294
Homocysteine, µmol.l^-1^	12.8±8.8	12.5±8.7	14.0±9.1	0.413
Previous stroke^§^, %	9.5	10.9	3.3	0.202
Previous AMI^§^, %	4.2	4.3	3.3	0.801
Previous CAD^§^, %	1.8	2.2	-	0.415
Sedentary^§^, %	60.7	61.6	56.7	0.616
Smoker^§^, %	37.3	36.5	41.4	0.621
klotho, ng.ml^-1^	1.2±0.6	1.2±0.6	1.2±0.8	0.743

Data are expressed as average±standard deviation for continuous parameters or relative frequencies for categorical features. Student’s t test or the chi-square^§^test were used. WC = waist circumference; HbA1c = glycated hemoglobin A1c; HOMA = Homeostatic model assessment; DM2 = type 2 diabetes mellitus; LDL-c = low density lipoprotein cholesterol; HDL-c = high density lipoprotein cholesterol, SBP = systolic blood pressure; DBP = diastolic blood pressure; SAH = systemic arterial hypertension; CRP = C-reactive protein; TSH = thyroid-stimulating hormone; AMI = acute myocardial infarction; CAD = coronary artery disease. Significance threshold set at *p*≤0.002 according to the Bonferroni correction.

**Table 2:** Correlation analyses of raw serum levels of C-reactive protein, homocysteine and Klotho across clinical, biochemical and healthcare features of the 168 older subjects at admission.

	Age	Gender^§^	SBP	DBP	SAH^§^	αSAH^§^
CRP	0.09; 0.242	0.23; 0.002^#^	-0.07; 0.399	-0.10; 0.195	0.09; 0.268	0.09; 0.238
Homocysteine	0.18; 0.022	-0.25; 0.001^£^	-0.10; 0.203	-0.18; 0.022	0.05; 0.544	0.07; 0.373
Klotho	0.11; 0.178	0.06; 0.424	-0.18; 0.025	-0.16; 0.041	-0.06; 0.473	-0.05; 0.538
	WC	Sedentary^§^	HbA1c	HOMA	DM2^§^	αDM2^§^
CRP	0.08; 0.289	0.23; 0.002^¥^	-0.07; 0.400	0.20; 0.010	0.05; 0.492	0.01; 0.873
Homocysteine	0.02; 0.791	0.11; 0.165	-0.02; 0.764	0.01; 0.933	0.09; 0.233	0.01; 0.884
Klotho	-0.05; 0.524	0.07; 0.375	-0.07; 0.353	-0.07; 0.404	-0.01; 0.898	0.03; 0.660
	TC	LDL-c	TGL	hyperlipidemia	HDL-c	αlipemia^§^
CRP	0.03; 0.722	-0.06; 0.433	-0.01; 0.887	0.03; 0.686	-0.03; 0.687	-0.01; 0.968
Homocysteine	-0.12; 0.119	-0.06; 0.474	-0.03; 0.672	-0.06; 0.476	-0.09; 0.233	0.06; 0.405
Klotho	-0.18; 0.021	-0.10; 0.204	-0.05; 0.537	-0.01; 0.935	-0.06; 0.444	-0.02; 0.802
	Drinker^§^	Smoker^§^	DIC	DIL	DIP	TEI
CRP	0.12; 0.108	0.13; 0.098	-0.05; 0.610	-0.04; 0.697	0.18; 0.050	-0.04; 0.673
Homocysteine	0.17; 0.025	0.12; 0.134	-0.13; 0.161	0.13; 0.179	0.07; 0.484	0.01; 0.924
Klotho	0.02; 0.790	0.14; 0.086	0.10; 0.277	-0.19; 0.045	0.12; 0.193	-0.20; 0.031

The Pearson’s and the Spearman’s^§^ correlation tests were used. For the latter, the presence or absence of a feature was represented by a 1 or a 0, respectively. Data are expressed in correlation indexes and significance level (*r*; *P*). α = use of therapeutic drugs for the condition; WC = waist circumference; SBP = systolic blood pressure; DBP = diastolic blood pressure; TC = total cholesterol; HDL-c = high density lipoprotein cholesterol; HOMA = Homeostatic model assessment; HbA1c = glycated hemoglobinA1c; SAH = systemic arterial hypertension; DM2 = type 2 diabetes mellitus; DIC = dietary intake of carbohydrates; DIL = dietary intake of lipids; DIP = dietary intake of proteins; TEI = total energy intake. The significance threshold was set at *p*≤0.002 after adjustment using the Bonferroni correction. Superscript #, £ and ¥ refer to effect sizes (*d*) and 95% confidence intervals (in parenthesis) of 0.23 (0.1; 0.4), -0.25 (- 0.4; -0.1) and 0.23 (0.1; 0.4), respectively.

**Table 3:** Correlation analyses of raw serum levels of C-reactive protein, homocysteine and Klotho across carriers and non-carriers of vascular events that occurred prior to admission in the study.

	Vascular events		
	Stroke	AMI	CAD
CRP †,‡	0.14; 0.066	0.04; 0.615	-0.06; 0.465
homocysteine †	0.12; 0.124	0.01; 0.902	0.01; 0.911
Klotho	0.07; 0.381	0.22; 0.006#	-0.13; 0.100

The Spearman’s and the Partial (for adjustments) correlation tests were used and were adjusted for

† gender and/or

‡ physical activity when appropriate. The presence or absence of a feature was represented by a 1 or a 0, respectively. Data are expressed as correlation indexes and significance levels (*r*; *P*). AMI = acute myocardial infarction; CAD = coronary artery disease. The significance threshold was set at *p*≤0.016 after adjustment using the Bonferroni correction. Superscript

# refers to an effect size of 0.22 and a 95% confidence interval of (0.1; 0.4).
